# Multi-annual time series operational data for a Vestas V52 wind turbine located in a coastal peri-urban environment in Ireland

**DOI:** 10.1016/j.dib.2023.109227

**Published:** 2023-05-12

**Authors:** Raymond Byrne, Paul MacArtain

**Affiliations:** Centre for Renewables and Energy, Dundalk Institute of Technology, Dublin Road, Dundalk, Co. Louth, Ireland. A91 K584

**Keywords:** Distributed wind, Peri-urban wind, SCADA data, Power performance, Real-world data

## Abstract

This long-term time series wind turbine operation dataset is from an 850 kW Vestas V52 wind turbine located in a peri-urban location in Ireland. The wind turbine has a hub height of 60 m and a rotor diameter of 52 m. The dataset ranges from 2006 to 2020 and contains the 10-minute raw data logged by the internal turbine controller system. It includes both external environmental measurements such as wind speed, wind direction and temperature, and wind turbine operating parameters such as rotor speed, blade pitch angle, generator speed and internal component operating temperatures. The data may be of interest for a variety of wind research areas including distributed wind energy, wind turbine ageing, technology improvements, design standards development and wind turbine energy performance in per-urban environments under various atmospheric conditions.

**Specifications Table**

**Table:** 

Subject	*Renewable Energy, Sustainability and the Environment*
Specific subject area	*Operational data of large scale wind turbines in peri-urban environments, relevant to industrial scale behind-the-meter wind applications*
Type of data	Table
How the data were acquired	The wind turbine is controlled by an internal SCADA system developed by Vestas. It consists of a number of microprocessors that monitors a range of turbine operational parameters including power output and wind information from a nacelle mounted Thies 2-D ultrasonic anemometer. Wind flow is sampled between 4 ultrasonic sensors at 50 Hz from which the 10-minutes wind speed and direction data averages are logged. It has a resolution of 0.1 m/s and 1^o^ in wind speed and direction respectively. The SCADA system stores 1 month of 10-minute data values, therefore external data storage is required to capture 10-minute data files on a long-term multi-annual basis.
Data format	Raw data in .csv format
Description of data collection	*10-minute average values are: mean wind speed and standard deviation, absolute wind direction and relative nacelle direction, rotor revs per minute, blade pitch angle, electrical power (including minimum and maximum power values), gear oil temperature, gear bearing and generator bearing temperatures, generator winding temperatures, internal nacelle temperature and the external ambient temperature.*
Data source location	· Dundalk Institute of Technology: · Dundalk: · Ireland: · GPS coordinates: 53.98352, -6.391390
Data accessibility	Repository name: Mendeley Data Data identification number: DOI:10.17632/tm988rs48k.2 Direct URL to data: https://data.mendeley.com/datasets/tm988rs48k
Related research article	R. Byrne and P. MacArtain, “The 15-year operational experiences of an 850 kW peri-urban wind turbine: Lessons learned from a behind-the-meter installation in Ireland,” Energy Sustain. Dev., vol. 70, pp. 342–360, 2022. https://doi.org/10.1016/j.esd.2022.08.011

## Value of the Data

• Long-term high-resolution time series data from an operating wind turbine in a peri-urban environment can help assess the factors the impact the lifetime operation of wind energy systems in such environments.
• Various stakeholders in the area of distributed wind in peri-urban environments may make more informed decisions to optimally deploy wind energy systems based on real-world operational data from an example case.
• Research studies in wind turbine performance, turbine aging, flow model development and verification as well as wind turbine design standard development can benefit from access to real-world operational data.

## 1 Objective

To date, very few studies have been published on energy performance of large scale wind turbines operating in peri-urban wind environments and the characteristics of the wind resource that affect an operating turbine's energy performance. Therefore, a better understanding of these types of projects would help ensure the future market success of this sector of the distributed wind industry. This dataset captures the real-world operation of a behind-the-meter deployed Vestas V52 wind turbine over a decade in the peri-urban environment of the Dundalk Institute of Technology (DkIT) campus in Ireland. The wind turbine has a 60 m hub height and a 52 m rotor diameter with a power rating of 850 kW. With an increased global focus on renewable and growing opportunities in wind energy, this dataset may be of interest to a number of stakeholders such as, prospective end-users, installers, planners, policy makers, researchers and project investors.

## 2 Data Description

The dataset consists of a single .csv file that contains multi-annual 10-minute time series raw Supervisory Control and Data Acquisition (SCADA) data as well as a data descriptor file. The raw data file is approximately 77 MB in size. The data acquisition period covers from the 30 January 2006 to 12 March 2020, representing the time period to from which data acquisition commenced up the beginning of the COVID-19 global pandemic. Ongoing data capture after this period became restricted and sparse due to limited site access and is not included here. Table 1 outlines the data channel column headings each of which has just over 653,000 data points. In a turbine faulted condition, the blade pitch angle “Pitch” is set to 86.6 degrees while it is typically less than 20 degrees in normal operation. This parameter can therefore be used to filter out times of normal turbine operation. It should be noted that a gearbox change out took place in the period from 04 October 2018 to 27 July 2019 for which there is no positive electrical power output recorded. The data descriptor file has the same information as Table 1.

**Table 1:** SCADA data parameter description.

Parameter	Description
Timestamps	*Time stamps in 10-minute intervals*
WindSpeed	Average 10-minute wind speed (m/s)*
StdDevWindSpeed	Wind speed standard deviation (m/s) in 10-minute period*
WindDirAbs	Average 10-minute absolute wind direction (deg) *
WindDirRel	*Average 10-minute relative direction of nacelle with respect to WindDirAbs (deg)*
Power	*Average 10-minute power output (kW)*
MaxPower	*Maximum 10-minute power output (kW)*
MinPower	*Minimum 10-minute power output (kW)*
StdDevPower	*Power output standard deviation (kW) in 10-minute period*
AvgRPow	*Average 10-minute reactive power output (kVAR)*
Pitch	*Average 10-minute blade pitch angle (deg)*
GenRPM	*Average 10-minute electrical generator revs per minute (RPM)*
RotorRPM	*Average 10-minute wind turbine rotor revs per minute (RPM)*
EnvirTemp	*Average 10-minute environmental temperature outside of nacelle (°C)*
NacelTemp	*Average 10-minute temperature inside nacelle space (°C)*
GearOilTemp	*Average 10-minute gearbox oil temperature (°C)*
GearBearTemp	*Average 10-minute gearbox bearing temperature (°C)*
GenTemp	*Average 10-minute generator temperature (not active-999)*
GenPh1Temp	*Average 10-minute generator phase 1 winding temperature (°C)*
GenPh2Temp	*Average 10-minute generator phase 3 winding temperature (°C)*
GenPh3Temp	*Average 10-minute generator phase 3 winding temperature (°C)*
GenBearTemp	*Average 10-minute generator bearing temperature (°C)*

* Measured by a 2-D ultrasonic Thies Clima anemometer that is located on the wind turbine nacelle

## 3 Experimental Design, Materials and Methods

**Fig. 1:**
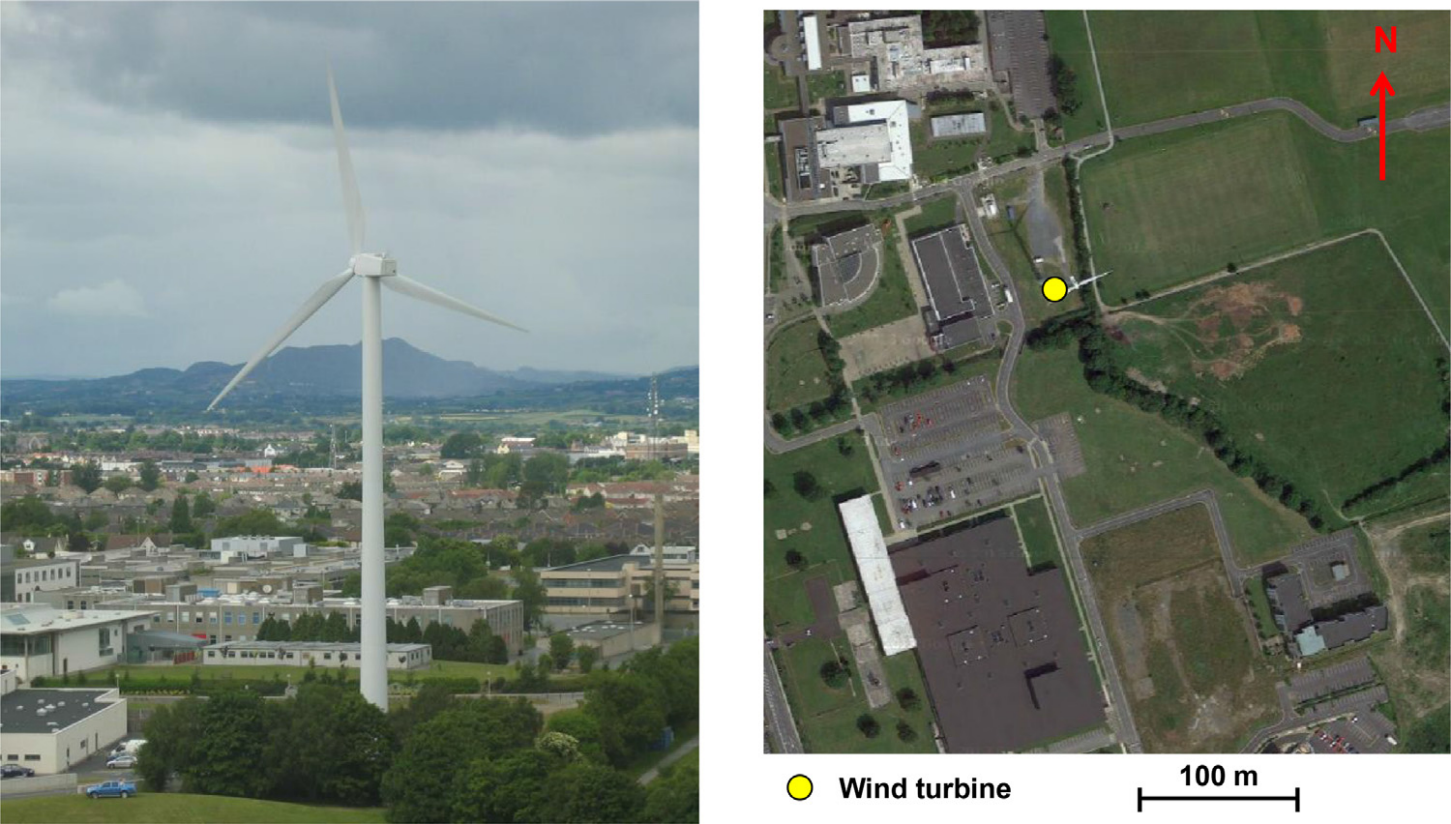
Wind turbine site.

The wind turbine is located on the campus Dundalk Institute of Technology (DkIT) in Ireland (co-ords: 53.983^o^, -6.392^o^) as shown in Fig. 1.

**Fig. 2:**
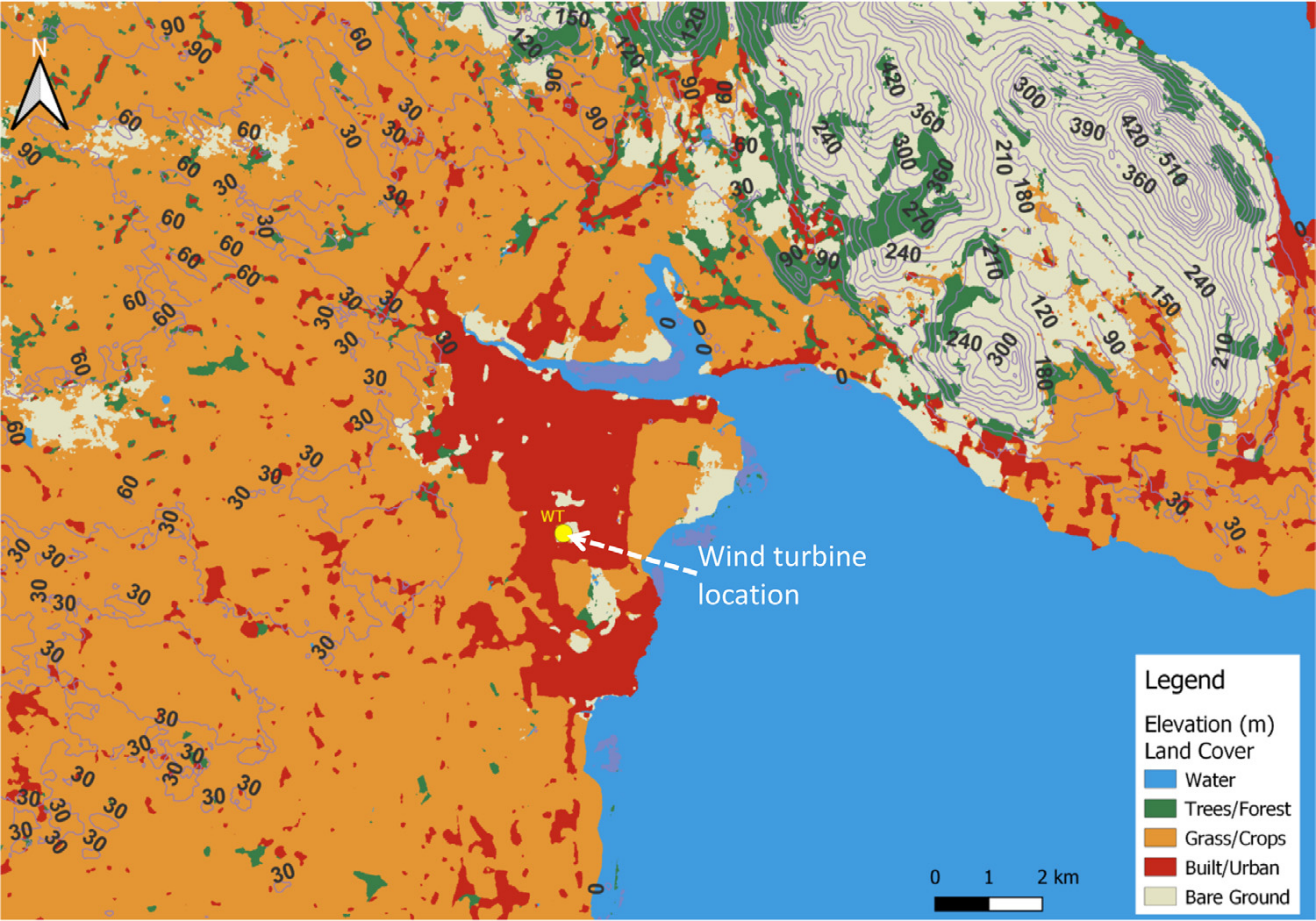
Elevation and land cover within a ∼ 20 km radius of site (Map created using Open Source QGIS [1]).

The turbine site has an elevation of ∼ 13 m above sea level. The surrounding rural terrain within a 20 km radius of the wind turbine site is low-lying agricultural land as shown by the map in Fig. 2, created using open source QGIS [1]. Elevation data is determined from Shuttle Radar Topography Mission (SRTM) data projected to the world geodetic system 1984 (WGS84) reference system and transformed to the universal transverse mercator (UTM) co-ordinates for Zone 29 [2]. Land cover is determined from the Sentinel-2 10 m land use/land cover time series of the world [3,4]. Large areas have elevations below 50 m with sparsely dispersed shelterbelts approximately 3 m high. It has elevations below 50 m with sparsely dispersed shelterbelts approximately 3 m high. The coast of Dundalk bay is approximately 3 km to the east of the site. To the north and northeast, approximately 7.5 km to 20 km away, there are hills that range in elevation from 75 m to 563 m. With respect to the turbine location, nearby obstacle features consist of various low-rise buildings at various distances from the wind turbine in each direction, within ∼ 1 to 2 km radius of the wind turbine location. The majority of the buildings below 20 m in height, along with one tall and narrow 47 m building approximately 335 m to the south-east of the turbine location.

All wind turbine functions are monitored and controlled by the Vestas Multi Processor (VMP) unit, that consists of a number microprocessor based control units, located inside the with turbine nacelle. It serves as a SCADA system that monitors and controls all wind turbine functions and the collection of multiple data parameters. Wind speed and direction are measured by a nacelle mounted, Thies 2-D ultrasonic, anemometer. It has a sampling rate of 50 Hz from which the 10-minutes data averages are logged. It has a resolution of 0.1 m/s and 1^o^ in wind speed and direction respectively. The SCADA system stores one month of 10-minute data internally, therefore, each month of 10-minute data was captured and stored externally in order to build up the long-term multi annual 10-minute data set. A variety of research studies to date have used all or parts of the data set [5], [6], [7], [8], [9], [10], [11], [12], [13], [14], [15].

## Ethics Statements

The authors comply with the ethical guidelines contained in Data in Brief's Guide for Authors. This work did not involve human subjects, animal experiments nor data collected from social media platforms.

## CRediT authorship contribution statement

**Raymond Byrne:** Conceptualization, Data curation, Investigation, Writing – original draft. **Paul MacArtain:** Funding acquisition, Project administration, Writing – review & editing.

## Declaration of Competing Interest

The authors declare that they have no known competing financial interests or personal relationships that could have appeared to influence the work reported in this paper.

## Data Availability

Vestas V52 Wind Turbine, 10-minute SCADA Data, 2006-2020 - Dundalk Institute of Technology, Ireland (Original data) (Mendeley Data).
